# Automatic Detection of Short-Term Atrial Fibrillation Segments Based on Frequency Slice Wavelet Transform and Machine Learning Techniques

**DOI:** 10.3390/s21165302

**Published:** 2021-08-05

**Authors:** Yaru Yue, Chengdong Chen, Pengkun Liu, Ying Xing, Xiaoguang Zhou

**Affiliations:** 1 School of Modern Post (School of Automation), Beijing University of Posts and Telecommunications, Beijing 100876, China; yyr@bupt.edu.cn (Y.Y.); pkl@bupt.edu.cn (P.L.); 2 School of Economics and Management, Minjiang University, Fuzhou 350108, China; 1133@mju.edu.cn; 3 School of Artificial Intelligence, Beijing University of Posts and Telecommunications, Beijing 100876, China; xingying@bupt.edu.cn

**Keywords:** atrial fibrillation, electrocardiogram, P-QRS-T, frequency slice wavelet transform, machine learning, Gaussian-kernel support vector machine

## Abstract

Atrial fibrillation (AF) is the most frequently encountered cardiac arrhythmia and is often associated with other cardiovascular and cerebrovascular diseases, such as ischemic heart disease, chronic heart failure, and stroke. Automatic detection of AF by analyzing electrocardiogram (ECG) signals has an important application value. Using the contaminated and actual ECG signals, it is not enough to only analyze the atrial activity of disappeared P wave and appeared F wave in the TQ segment. Moreover, the best analysis method is to combine nonlinear features analyzing ventricular activity based on the detection of R peak. In this paper, to utilize the information of the P-QRS-T waveform generated by atrial and ventricular activity, frequency slice wavelet transform (FSWT) is adopted to conduct time-frequency analysis on short-term ECG segments from the MIT-BIH Atrial Fibrillation Database. The two-dimensional time-frequency matrices are obtained. Furthermore, an average sliding window is used to convert the two-dimensional time-frequency matrices to the one-dimensional feature vectors, which are classified using five machine learning (ML) techniques. The experimental results show that the classification performance of the Gaussian-kernel support vector machine (GKSVM) based on the Bayesian optimizer is better. The accuracy of the training set and validation set are 100% and 93.4%. The accuracy, sensitivity, and specificity of the test set without training are 98.15%, 96.43%, and 100%, respectively. Compared with previous research results, our proposed FSWT-GKSVM model shows stability and robustness, and it could achieve the purpose of automatic detection of AF.

## 1. Introduction

The mortality rate of cardiovascular diseases (CVDs) has been extremely high [1,2,3]. In fact, about 422.7 million people in the world suffered from CVDs of different degrees and types, and about 17.92 million patients died due to CVDs in 2015 [1]. Particularly, atrial fibrillation (AF) is a major CVD, affecting over 33.5 million individuals worldwide [4]. AF is also the most common persistent cardiac arrhythmia [5]. The heart rate is as high as 100–160 beats/min when AF attacks. The electrocardiogram (ECG) signal shows irregular R-R intervals (RRIs), disappeared P wave, and appeared F wave. AF has strong associations with other CVDs, such as myocardial infarction (MI) and chronic heart failure (CHF) [6]. Moreover, stroke patients with the highest mortality rate are more likely to suffer from AF, which is up to 5 times more likely than the general population [7]. Therefore, automatic detection is very helpful for the early treatment of AF and prevention of related complications.

Generally, an ECG signal is used to detect AF. ECG signal polluted by multiple interferences has non-stationary characteristics, including baseline drift, electromyogram (EMG) interference, power frequency interference, electrode contact noise, human motion noise, and instrument noise [8]. It is difficult to express comprehensive information effectively if only using time-domain or frequency-domain analysis methods. Hence, time-frequency technology is created to show time-domain and frequency-domain information at the same time.

The Fourier transform (FT) was proposed to solve the problem of frequency domain analysis in 1807. Gabor added a window function based on FT and proposed short-time Fourier transform (STFT) in 1946. STFT avoided the deficiency of local expression ability of the FT method in the time-frequency domain. However, it was difficult to obtain the ideal multi-resolution due to the fixed window function. Wigner proposed the distribution function, namely, Wigner distribution (WD), in 1932. WD was applied to the field of signal analysis and processing by Ville in 1947, and then developed into a representative bilinear time-frequency analysis method, known as Wigner-Ville distribution (WVD). It had a good time-frequency resolution in [9]. In fact, results of WVD will be affected by false information caused by cross interference terms. Wavelet transform (WT) was proposed by Morlet in the 1980s. It was introduced into the field of signal processing by Meyer later. A scalable window function was obtained by selecting the scale factor and shift factor. The ratio of the bandwidth remained unchanged so that the window function could automatically adapt to the change of the frequency component of the signal. The window of short time was used for the high frequency component, while the window of long time was used for the low frequency component. WT had good localization characteristics. In recent years, various WTs have been derived from preprocessing ECG signals, mainly including continuous wavelet transform (CWT), discrete wavelet transform (DWT), empirical wavelet transform (EWT), stationary wavelet transform (SWT), double-tree complex wavelet transform, different wavelet packets and WT combining different wavelet bases with threshold method [10,11,12]. Therefore, the frequency slice wavelet transform (FSWT) was proposed by Yan—who combined the characteristics of STFT, WVD, and WT [13]. FSWT introduced the frequency slice function, inherited the characteristics of the wavelet function, and made the traditional FT method controllable [14].

Meanwhile, FSWT was widely used in vibration signal (including beam structure, overvoltage, bearing, explosion, and seismic), biological signal, and noise detection. For instance, Liu et al. [15] located the damage of beam structure using the FSWT method, which improved the accuracy of damage location. Zhang et al. [16] extracted features of overvoltage waveforms using FSWT technology, and then used stacked sparse autoencoders to classify overvoltage effectively. Luo et al. [17] introduced the bounded adaptive frequency slice function as the dynamic frequency filter to improve the FSWT for ECG, PPG, and PCG physiological signals. In summary, the studies indicated that the FSWT could effectively display the time-frequency domain information. Thus, it was applied to the feature extraction for automatic detection of AF.

Machine learning (ML) algorithms have strong interpretability, stability, and robustness. They are widely used in practice because ML models consume relatively less energy and can quickly calculate the detection results. ML can be applied to wearable and portable ECG monitoring devices, including smartphones, watches, glasses, clothing, etc. [18,19,20]. For example, Liu et al. [18] extracted the power feature, spectrum feature, entropy feature, RRIs feature, and P-wave feature of the ECG signals collected from AliveCor portable ECG equipment. They then used support vector machine (SVM) algorithm to identify AF. The F1 value of the training set and the test set were 0.84 and 0.80, which conquered the results of many deep learning algorithms. Zhang et al. [19] extracted power spectrum, baseline drift, amplitude difference, and other time-domain features from mobile phones-collected ECG to create the feature matrix. Then, the feature matrix was classified by KSVM and GA algorithms. The accuracy of the training sets A and B was 94.0% and 91.8%, respectively. Chon et al. [20] used smartwatches to detect AF in cardiac rehabilitation, and the diagnosed result was 66% of patients. The sensitivity and specificity of AF compared to lead 12 were 93% and 84%, respectively.

Furthermore, Xin et al. [21] extracted the multi-scale Rényi entropy of HRV from the MIT-BIH PAF Prediction Challenge Database and used the SVM classifier to attain the accuracy of 92.48% and the specificity of 91.76%. Colloca et al. [22] used SVM to classify AF with 10 RR features. The sensitivity of the training set MIT-BIH Atrial Fibrillation Database (AFDB) was 99.07%. The specificity of the test sets MIT-BIH Normal Sinus Rhythm Database (NSRDB), and MIT-BIH Arrhythmia Database were 99.72% and 99.70%, respectively. Kumar et al. [23] used flexible analytic wavelet transform (FAWT) to decompose ECG signals of 1000 sample length, and calculated logarithmic energy entropy and permutation entropy for the obtained sub-band. Afterward, they used random forest (RF) to classify AF. The results of logarithmic energy entropy were superior. Accuracy, sensitivity, and specificity of classification were 96.84%, 95.8%, and 97.6%, respectively. Moreover, Kennedy et al. [24] analyzed the RRIs with four R-R irregular measurements: The coefficient of sample entropy (CoSEn), the coefficient of variance (CV), root mean square of the successive difference (RMSSD), and median absolute deviation (MAD). RF and k-nearest neighbor (KNN) were used for training. The sensitivity and specificity of the RF were 92.8% and 98.3%, respectively. To sum up, time domain, frequency domain, and nonlinear analysis methods were utilized in [18,19,21,22,23,24], and then ML techniques were used to identify AF.

Besides, the authors of [25,26] combined atrial activity with other methods, and used ML algorithms to detect AF. Bruun et al. [25] combined atrial activity and heart rate variability (HRV) as extracted features, and used the integration algorithm of the bagged tree to classify AF. The sensitivity, specificity, and accuracy of AF were 96.51%, 99.19%, and 98.22%, respectively. Babaeizadeh et al. [26] combined the Markov model method and atrial activity analysis, extracted three features, including R-R Markov score, P-R interval variability, and P-wave morphological similarity measurement. Next, they used the decision tree (DT) algorithm for classification. The sensitivity and specificity were 94% and 99%. In addition, Zhao et al. [27] used the modified FSWT to convert the one-dimensional ECG signals collected by wearable devices into the two-dimensional time-frequency diagrams, and then fed them into the convolutional neural network (CNN) for tri-classification, achieving an accuracy of 86.3%. The accuracy was not high enough to be improved.

As described above, these experimental results of AF detection based on ML algorithms were excellent, which indicated that ML techniques were robust and suitable for automatic detection and recognition of AF. Therefore, we combined the whole P-QRS-T waveform generated by atrial activity and ventricular activity as detected features, and then used the advanced and sophisticated ML techniques for classification so far.

In this paper, data is derived from the MIT-BIH AFDB. Time-frequency diagrams of short-term AF signals and normal ECG signals were obtained by the FSWT technology. Subsequently, the two-dimensional (2D) time-frequency matrices were transformed into the one-dimensional (1D) feature vectors. Finally, ML algorithms were used to classify AF/non-AF. Among them, the 10-fold cross-validation method was used to evaluate and select the trained classification models. The stability and generalization performance of the models were evaluated by evaluation indexes of accuracy, sensitivity, and specificity. Although the results of previous studies have achieved good performance, the models of FSWT-ML have two advantages. The first is to extract and analyze the characteristics of two-dimensional time-frequency ECG signals containing several continuous waveforms of P-QRS-T; the second is to train the FSWT-GKSVM model with better performance from a variety of ML models. This model is easier to achieve the automatic detection of short-term AF segments.

This paper is organized as follows. In Section 2, the MIT-BIH Atrial Fibrillation Database and the preprocessing of ECG signals are described. In Section 3, a technique combining FSWT and ML algorithms to detect short-term AF segments is proposed. Moreover, indicators of performance evaluation are introduced. In Section 4, the experiments are designed to find the best performance of the proposed model and discussed the effects of different fragment lengths and different ML algorithms. Finally, the conclusions are provided in Section 5.

## 2. Description of Data

### 2.1. Data Source

**Figure 1:**
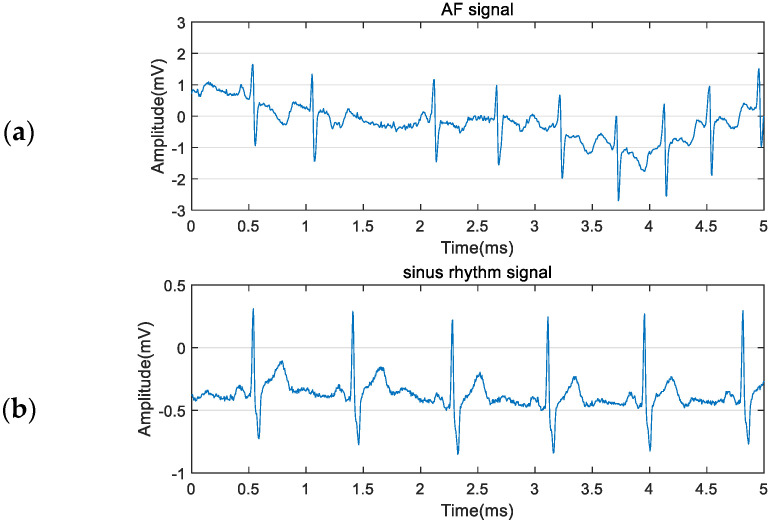
(**a**) AF recording and (**b**) Sinus rhythm recording.

The MIT-BIH Atrial Fibrillation Database (MIT-BIH AFDB) of PhysioNet was exploited in this study [28]. This database contains long-term ECG recordings from 25 subjects. Most of AF is paroxysmal atrial fibrillation (PAF). There are four types of manual rhythm annotations, which are AFIB (atrial fibrillation), AFL (atrial flutter), J (AV junctional rhythm), and N (used to indicate all other rhythms), respectively. We choose to study the AF signals annotated by AFIB rhythms. To maintain the balance of data categories, we only selected normal ECG signals except for AF signals. Fragments of the AF signal and the normal ECG signal are shown in Figure 1. As 00735 records and 03665 records are represented by the rhythm and unchecked beat annotation, only the remaining 23 records were used. Every recording includes two types of ECG signals sampled at 250 Hz with 12-bit resolution over a range of ±10 millivolts for up to 10 h. The recording numbers of specific ECG signals are shown in Table 1.

### 2.2. Data Preprocessing

Preprocessing of data plays a very important role in overall work. If it is not handled properly, it will directly affect the following work and the results of classification. Hence, we divide the process of data preprocessing into three steps as follows.

**Figure 2:**
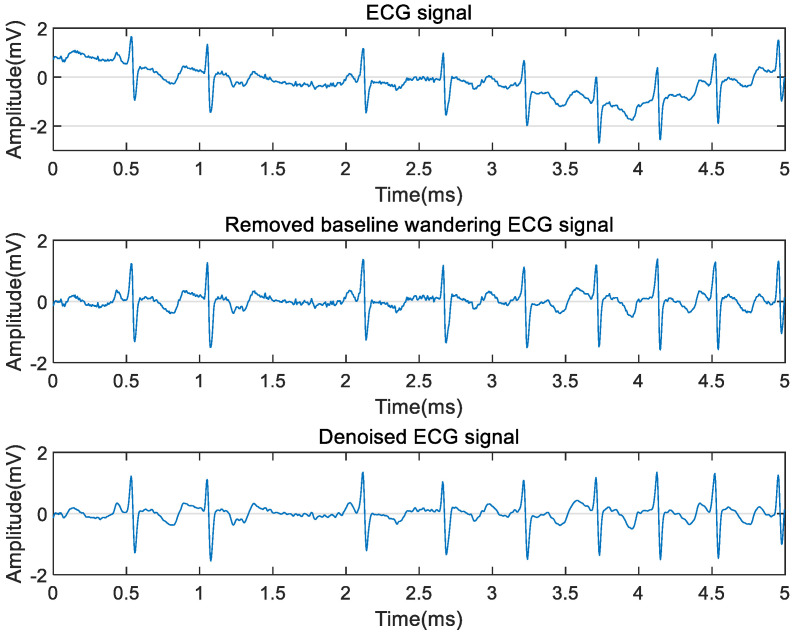
The implementation process of ECG signal denoising.

Firstly, baseline wandering and noises were removed from the contaminated ECG signals. The polynomial fitting method was used to remove the baseline drift. In the beginning, the least square method was used to fit the original ECG signals. Then, the trend term was obtained by using the minimum standard of the sum of squares of the difference between the fitting curve and the actual ECG signal. The order of removing the trend term was 13. Lastly, the ECG signal without the baseline drift was obtained by removing the trend term. On this basis, Savitzky-Golay (SG) filter was used to smooth the ECG signal. The appropriate window length and fitting order should be selected. The window length should not be too large and should be odd, and the parameter selection can be seen in [29]. In this study, the window length was 9, and the order was 3. This combined approach decreased the interference of non-stationary noise, reduced the complexity of ECG signal, and highlighted the characteristic information of waveforms. The specific implementation process is shown in Figure 2.

**Figure 3:**
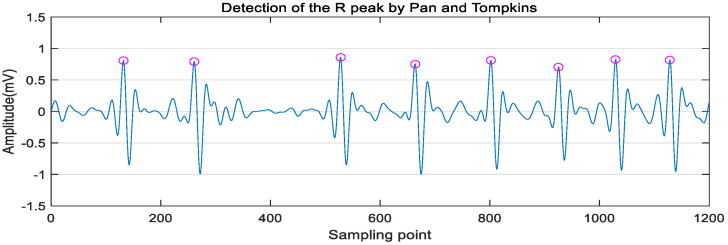
Detection of R peaks by Pan-Tompkins algorithm.

Thereafter, Pan-Tompkins (PT) algorithm was used to extract R peaks from the denoised ECG signal. Its steps mainly included differentiation, square, and sliding window integration to detect R peaks, as shown in Figure 3. The DC component of the input signal was removed by the derivative filter, and the slope of the waveform was enhanced at the same time. The square filter made the sample value positive and further enhanced the slope of the ECG waveform. The output ECG waveform was smoothed by an integral filter. Adaptive thresholds were used to search R peaks from pure ECG signals. The setting of parameters can be referred to [30].

**Figure 4:**
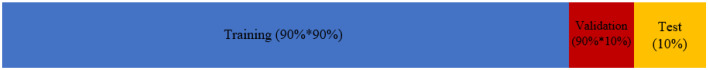
The percentage of ECG segments used for training, validation, and test.

Finally, short-term AF signals and normal ECG signals of different lengths were separated. In the experiment, we segmented the ECG signals with different lengths at 1 s, 2 s, 3 s, 4 s, and 5 s. Most ECG recordings from MIT-BIH AFDB are PAF signals, and the number of normal ECG signals is much larger than that of AF signals. Therefore, the number of the two types of ECG signals was balanced. The ratio of the training set and testing set was 9:1, which was randomly divided according to the stratified sampling method. The probability ratio of the number of AF signals and the number of normal ECG signals was consistent between the training set and the test set. The proportions of the training set and test set at different durations remained the same. The 10-fold cross-validation method was used for model selection and hyperparameters adjustment, and the percentage of ECG segments used for training, validation, and testing was shown in Figure 4. The number of specific ECG fragments is shown in Table 2.

## 3. Methods

### 3.1. Frequency Slice Wavelet Transform

FSWT technology was used for the time-frequency analysis of the preprocessed ECG signals. The principle of FSWT is as follows.

Assuming that $\hat{f}\left(u\right)$ is the FT of f(t), the representation of FSWT in the frequency domain is shown in Equation (1).

(1) $$W_{f}\left(t,\omega ,\sigma \right)=\frac{1}{2\pi }\int_{-\infty }^{+\infty }\hat{f}\left(u\right){\hat{p}}^{*}\left(\frac{u-\omega }{\sigma }\right)e^{iut}du,$$

where $\hat{p}\left(\omega \right)$ is the frequency slice function. t and ω are observation time and observation frequency, respectively. “*” stands for conjugate operation. σ is a regulator, and can be set as a constant or a function of ω, t and u. According to principle of WT, let σ be ω/k(k>0). Therefore, Equation (1) can be expressed as follows:

(2) $$W_{f}\left(t,\omega ,\sigma \right)=\frac{1}{2\pi }\int_{-\infty }^{+\infty }\hat{f}\left(u\right){\hat{p}}^{*}\left(k\frac{u-\omega }{\omega }\right)e^{iut}du,$$

where k is independent of u and ω. It is called time-frequency resolution factor, which is used to adjust the sensitivity of time-frequency transformation. We set it as a function of the observation time and the observation frequency.

Common functions of frequency slice are included in Equation (3).

(3) $$\hat{p}\left(\omega \right)=\{\begin{array}{c} e^{-\left|\omega \right|}\\ e^{-\omega^{2}/2}\\ \frac{1}{1+\omega^{2}} \end{array}.$$

Thus, FSWT realized the decomposition of ECG signal in the time domain and frequency domain. Due to the different selection of the frequency slicing function, and the fact that the time domain and frequency domain of the ECG signal were interdependent, various forms of inverse transformation were generated to reconstruct the ECG signal. Assuming $\hat{p}\left(0\right)=1$, the original f(t) signal was reconstructed as follows:

(4) $$f\left(t\right)=\frac{1}{2\pi }\int_{\omega_{1}}^{\omega_{2}}\int_{t_{1}}^{t_{2}}W_{f}\left(\tau ,\omega ,\sigma \right)e^{i\omega \left(t-\tau \right)}d\tau d\omega .$$

According to Equation (4), the reconstructed ECG signal could be independent of the frequency slice function, but related to the regulator factor. By adjusting any selected values of $\omega_{1}$, $\omega_{2}$, $t_{1}$, $t_{2}$ in the time-frequency intervals, the ECG signal under different observation time and frequency ranges could be obtained.

**Figure 5:**
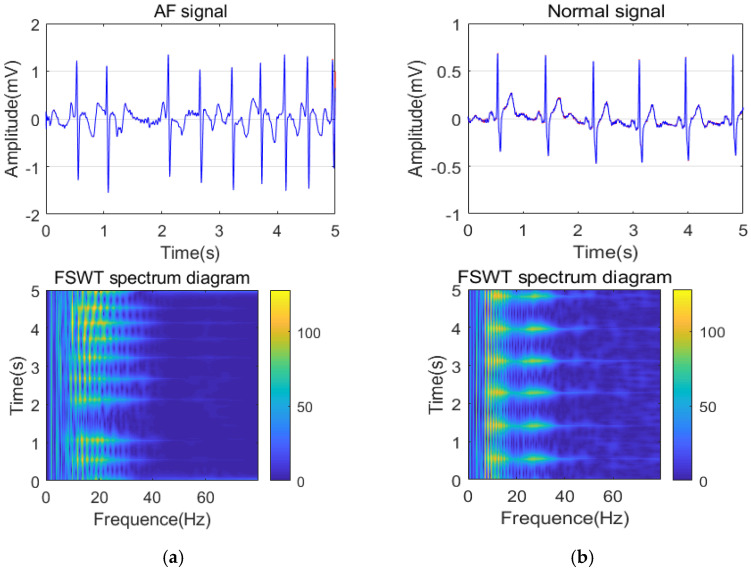
(**a**) Time-frequency spectrum of FSWT for the AF signal; (**b**) Time-frequency spectrum of FSWT for the normal ECG signal.

FSWT method was performed on each sample of preprocessed ECG signals to obtain 2D time-frequency spectrum diagrams of AF signals and normal ECG signals, as shown in Figure 5. The 2D time-frequency diagrams could display time and frequency information at the same time. The different colors in 2D time-frequency spectrum diagrams were represented by 0.255. 0.255 made up the 2D time-frequency matrices. ML classifiers were used to identify and classify AF segments. Considering that the 2D time-frequency matrices were too large and the calculation time was too long, we used an average slider to process each time-frequency matrix. The average slider consists of 1. The resultant processed matrix was flattened to obtain a 1D feature vector. Therefore, each feature vector was composed of different values from 0 to 255. Each value corresponds to the result of each convolution. The obtained 1D feature vectors were used as the ML models’ input. It was not convenient for practical application. In experiments, we set the observation frequency from 0 to 80 Hz, and the observation time was 1 s, 2 s, 3 s, 4 s, and 5 s, respectively. For example, when the observation time was set to 5s, the time-frequency matrix was 1250 × 400. A 10 × 5 average slider was used to change the time-frequency matrix into a 125 × 80 matrix, and then it was flattened to a row vector containing 10,000 feature points. Hence, all 2D time-frequency matrices were transformed into the data set of 1D feature vectors.

### 3.2. Machine Learning Classifiers

ML techniques were particularly suitable for analyzing medical data features and diagnosing medical problems [31], and thus, were used to identify and detect ECG signals in this work.

#### 3.2.1. Support Vector Machine

The support vector machine (SVM) was proposed to solve binary classification. Characteristics of the input are mapped into a high-dimensional or even infinite dimensional feature space in a way. It converts the linearly indivisible problem into linearly divisible. The hyperplane is constructed. Moreover, it is very suitable for small samples, nonlinear and high-dimensional pattern recognition.

As an important factor in SVM, kernel function mainly includes linear kernel, Gaussian kernel, and polynomial kernel. In fact, SVM is a special kind of kernel method. It is worth remarkable that the Gaussian kernel is usually better than the linear kernel for nonlinear data distribution. In this study, the Bayesian optimizer was used to optimize the parameters of Gaussian-kernel SVM (GKSVM). The number of iterations was set to 30, and the optimization criterion was the minimum classification error. When the number of iterations was 23, the accuracy of the verification set was optimal. At the same time, the optimized hyperparameters were that the Gaussian-kernel scale was 979.08, and the box constraint level was 248.68.

#### 3.2.2. K-Nearest Neighbor

The K-nearest neighbor (KNN) is a non-parametric statistical algorithm that classifies similar samples into a category in feature space. The training process is non-explicit and only needs to store all the training samples. It is a lazy learning method. During the test, k nearest neighbors of the test samples were found in the training samples.

In our classification task, when the number of iterations was 24, the accuracy of the verification set was optimal. The samples of the test set were classified as the category with the highest vote among the nearest neighbor samples of 1. The optimization hyperparameters were the correlation of distance measurement and the anti-distance of distance weight.

#### 3.2.3. Bagged Tree

The aggregate base classifier of bagging was used for AF classification. When predicting the samples of the test set in the AF classification task, bagging passed the samples to the base classifier, collected the output, and voted on the markers. The winning markers were regarded as the prediction results. In addition, a prediction result was selected at random if there was a tie. Broadly speaking, the more unstable the learner was selected, the more obvious the effect would be improved for selecting a bagging learner. Furthermore, the experimental results of researchers showed that the performance of bagging using decision stumps was not as strong as that of bagging using decision trees, and the predicted effect of its ensemble model would eventually converge with the increase of ensemble scale. Therefore, decision trees were used as the base classifier in bagging.

#### 3.2.4. Decision Trees

Decision trees (DTs) consist of a series of tree-shaped decision tests organized in a divide-and-conquer manner. There is a feature test on each non-leaf node. The data in the node are divided into different subsets according to different feature values in the feature test. Each leaf has a category flag, and each example that falls on this leaf is set to this category flag. During the test, the samples would reach the leaf node through a series of feature tests from the root node, and then get the test results. Moreover, the Gini coefficient was used for the segmentation criteria of DTs. It could deal with numerical characteristics so that it was more commonly used. Evaluating and selecting each value of numerical features was used as a split point, which accordingly divided the data set into two subsets, one part containing the samples that were larger than the split point, and the other part containing the remaining samples. Furthermore, the performance of DTs was compared with that of the bagged tree.

#### 3.2.5. Naive Bayes

Naive Bayes classifier assumes that given class markers and features are independent of each other. It calculates the proportion of each feature value within each class. This avoids the estimation of joint probability. In the training set, the naive Bayes method estimates the probabilities for all categories and the joint probability distributions for all features. During the testing process, the naive Bayes selected the category markers of the test samples by maximizing the posterior probability among all the category markers. In addition, the naive Bayes model was trained as the contrast group, since the Bayesian optimizer was used to optimize SVM and KNN.

### 3.3. Performance Evaluation

SVM, KNN, bagged tree, DT, and naive Bayes in ML algorithms were used to carry out the one-dimensional feature vectors of the training data and test data, respectively. During the training process, a 10-fold cross-validation method was used to evaluate and select the model. Accuracy, sensitivity, and specificity were used to evaluate the generalization performance of the test set as follows:

(5) $$\mathrm{Accuracy}=\frac{\mathrm{TP}+\mathrm{TN}}{\mathrm{TP}+\mathrm{FP}+\mathrm{TN}+\mathrm{FN}}\;,$$

(6) $$\mathrm{Sensitivity}=\frac{\mathrm{TP}}{\mathrm{TP}+\mathrm{FN}}\;,$$

(7) $$\mathrm{Specificity}=\frac{\mathrm{TN}}{\mathrm{FP}+\mathrm{TN}}\;,$$

where TP is the number of AF signals predicted to be AF signals, FP is the number of normal sinus signals predicted to be AF signals, TN is the number of normal sinus signals predicted to be normal sinus signals, FN is the number of AF signals predicted as normal sinus signals.

## 4. Results and Discussion

### 4.1. Influence of Denoising Process on Generalization Performance

First, we studied the influence of denoising on classification performance in preprocessing. On the 3 s time-scale, the original ECG signals and denoised ECG signals were, respectively, used as training sets, and then SVM, KNN, bagged tree algorithms were used for training. Then test sets for the original signals and denoised signals were tested by SVM, KNN, bagged tree models to get three performance metrics, including accuracy, sensitivity, and specificity. The experimental results are shown in Table 3.

The performance metrics of the test set using three ML algorithms are improved from Table 3. In fact, the accuracy of denoised signals is increased on average by 4.271% compared with the accuracy of original signals. The sensitivity of denoised signals is improved by 4.763% more than the sensitivity of original signals. The specificity of denoised signals is 3.782% higher than that of original signals. In addition, the accuracy of the training set will be very high even if the training set is not denoised. Therefore, the average growth rate of 1.692% is not a small change. To sum up, it is necessary to carry out an effective denoising process in the preprocessing as noise has an obvious influence on short-term ECG signals. This combined denoised approach could reduce the interference of noise, and highlight the characteristic information of waveforms.

### 4.2. Influence of Time-Scale on Generalization Performance

As shown in Table 3, the overall performance of the three assessment criteria of the optimized SVM model is relatively higher. Therefore, an optimized SVM algorithm was used to study the influence of time-scales on the generalization performance. Table 4 shows the performance evaluation results of test sets in different time-scales based on the optimized SVM algorithm.

The performance metrics of 1 s and 2 s are relatively smaller in different time-scales from Table 4, while the overall performance of 3 s, 4 s, and 5 s is relatively balanced. In particular, the difference between the three metrics of 4 s and 5 s is subtle, both of which are more than 90%. In addition, poor performance indicators are caused by a too small timescale, which leads to the failure of the model to learn the correlation characteristics between adjacent heart beats. Once the time-scale is up to 3 s, the accuracy and sensitivity are increased to 92.59% and 96.43%. Moreover, when the temporal scale increases to 5 s, although the value of sensitivity does not continue to increase, the accuracy, sensitivity, and specificity of the test set show a balanced overall performance, all of which are higher than 92%. The difference between the time-scales of 4 s and 5 s is that one is missed detection, and the other is a misjudgment. Our research focuses more on detecting sensitivity, so we are more inclined to choose the one with greater sensitivity. Furthermore, considering that the amount of data will increase with the increase of time-scale, the running time and complexity of the model are proportional to the size of the amount of data. Accordingly, it is more appropriate to select a 5 s time-scale for FSWT according to the experimental results, and the generalization performance is more balanced. In the 5s time-scale, the relevant information of adjacent waveforms could be fully displayed.

### 4.3. Influence of Different ML Techniques on Generalization Performance

According to the data analysis from Table 4, we used the 5s temporal scale to study the influence of the ML algorithms on the generalization performance. Table 5 shows the performance metrics results of the dataset on the 5 s time-scale based on different ML algorithms.

As shown in Table 5, three algorithms of SVM, KNN, and bagged tree have similar performance in each index, but the sensitivity of the optimized SVM model is the highest, and the accuracy of the validation set and training set is also excellent. The experiments from Table 4 and Table 5 jointly show that the optimized SVM model is a suitable classification model for our AF dataset.

### 4.4. Influence of Average Sliding Window on Generalization Performance

The size of the characteristic quantity is determined by the size of the average sliding window. A small amount of data with excellent generalization performance makes the practical application possible, the sizes of the average sliding window were discussed. The accuracy of feature sets was obtained by using different average sliding windows based on the optimized SVM algorithm from Table 6.

As shown in Table 6, when the average sliding windows are 5 × 20 and 25 × 10, the performance indicators of the training set and test set are relatively excellent. Table 7 shows the details of the performance metrics of the validation set, training set, and test set when the average sliding windows are 5 × 20 and 25 × 10. In brief, a 5 × 20 average sliding window is selected to transform the two-dimensional feature matrices obtained by FSWT based on the results from Table 6 and Table 7.

### 4.5. Comparison of Experimental Results of AF Detection

Table 8 summarizes the relevant research results for AF classification and recognition, mainly including the author, database, method, and three evaluation indicators of accuracy, sensitivity, and specificity. In addition, the database must contain MIT-BIH AFDB, and methods include machine learning algorithms, deep learning algorithms, or morphological analysis methods.

In [32], Asgari et al. used stationary wavelet transform combined with SVM to detect AF in short-term ECG signals. The sensitivity and specificity of the method reached 97.0% and 97.1%, respectively. The area under the Receiver Operative Characteristics (ROC) curve with two-fold cross-validation was 99.5%. This method eliminated the need for beat-dependent detection and wave peak detection (P peak or R peak). In addition, Anderson et al. [33] used SVM to classify AF based on sample entropy, sample entropy coefficient, Shannon entropy, RMS of continuous difference, and normalized RMS for 60, 100, and 300 beats. At the same time, SVM was used to classify AF based on the two features of peak average power ratio and logarithmic energy entropy extracted from the 2-stage stationary wavelet transform coefficients for 10 s, 15 s, and 30 s ECG signals. The research process of [32] was restored as a data-driven method. As presented by the authors of [32,33], features were extracted in the time domain and frequency domain, together with various entropy analysis methods. According to the results of the study, SVM had an excellent effect on the identification of AF for the characteristics of ECG signals.

Moreover, bagged tree and DT algorithms were also suitable for short-term ECG signals detection according to [25,26] so that they were trained by us. Kumar et al. used the RF algorithm to train long-term ECG signals. Although their research was very good, the results were slightly lower than our test results. We also used the RF algorithm to train our data, but the results were not very ideal. The RF model was potentially more suitable for long-term ECG signals.

Besides, Faust et al. [34] designed a six-layer bidirectional LSTM network to classify AF/non-AF with ECG signals of 100 beats, and the accuracy, sensitivity, and specificity of the model reached 98.51%, 98.32%, and 98.67%, respectively. Moreover, based on CNN, Dang et al. [35] added a bidirectional LSTM network to form a nine-layer deep neural network, and classified AF for 100 consecutive R peak sample points. The accuracy, sensitivity, and specificity of the model were 96.59%, 99.93%, and 97.03%. The accuracy of the training set and validation set were 99.94% and 98.63%. Compared with [34,35], the sensitivity of our study was 1.89% and 3.5% lower than that of the deep learning model, respectively. In addition, other performance indicators were similar. The deep neural network was applied to perform end-to-end feature extraction and classification from the 1D feature vectors or the 2D feature matrices [34,35,36,37,38]. It provided a new idea for the further study of automatic detection of AF.

Meanwhile, Wei et al. [37] used a recursive complex network to construct the synchronization features of a single beat independent of the R peak, and then designed a six-layer CNN combined with a voting algorithm for AF classification. The sensitivity, specificity, and accuracy reached 94.28%, 94.91%, and 94.59%, respectively. Although their research relied on beat detection, it did not depend on R peak detection, which purely analyzed atrial activity. Notably, the T wave was more prominent due to avoid the influence of the QRS wave group in this method, which provided a research idea for the study of myocardial infarction and myocardial ischemia.

Furthermore, Xu et al. [38] converted the 1s ECG fragments into the two-dimensional time-frequency images by using improved FSWT, which were input to 12-layer CNN for feature extraction and classification. The accuracy reached 81.07% and 84.85%, respectively, in the test set and the test set that eliminated poor quality ECG signals. Compared with our study, it directly used the two-dimensional time-frequency diagrams as the input of CNN, and our study converted the two-dimensional time-frequency matrices into the one-dimensional feature vectors. ML algorithms could be used for detection and recognition. Furthermore, the 1 s ECG segments lacked the relevant information of adjacent waveforms compared with the 5 s ECG segments from the perspective of better experimental results.

In short, our work is more comprehensive, combining atrial activity and ventricular activity and covering at least two cycles of cardiac activity. The ML algorithms are robust and more suitable for the automatic detection of AF. Admittedly, there is a limitation to our study. The restriction is that other databases are not integrated in addition to MIT-BIH AFBD. Although evaluation indicators of the training set, validation set, and test set performed excellently, the problem of using a single database cannot be ignored. Therefore, the next research combined with other databases will be conducted for a more depth-in study to achieve better generalization performance of the model. Moreover, we will try to use other methods to evaluate effective features for further improving the accuracy of ML models, such as the principal component analysis method [39].

## 5. Conclusions

In this paper, we proposed automatic detection of short-term AF segments mainly used the algorithm combined with FSWT and the optimized GKSVM algorithm, which comprehensively took advantage of the time-frequency domain characteristics of ECG signals and evaluated the occurrence of AF for short-term ECG signals overall. The experimental results were excellent in the 5 × 20 average sliding window, and the accuracy, sensitivity, and specificity of the test set reached 98.15%, 96.43%, and 100%, respectively. Moreover, the accuracy of the training set and validation set reached 100% and 93.4%, respectively. Automatic detection of short-term AF fragments can solve the problem of the high public health burden of CVDs in the world, and can also meet the needs of people in areas with less developed medical resources for AF monitoring.

## Figures and Tables

**Table 1:** A list of recording numbers using the database.

Sequence	Recording Numbers					
1	04015	04043	04048	04126	04746	04908
2	04936	05091	05121	05261	06426	06453
3	06995	07162	07859	07879	07910	08215
4	08219	08378	08405	08434	08455	

**Table 2:** The number of ECG segments at different durations.

Duration	Training Set	Testing Set
1 s	2410 × 250	270 × 250
2 s	1928 × 500	216 × 500
3 s	964 × 750	108 × 750
4 s	964 × 1000	108 × 1000
5 s	482 × 1250	54 × 1250

**Table 3:** Comparison of performance metrics of training set and test set of original ECG signals and denoising ECG signals using ML algorithms on the 3s time-scale.

Models	Signal Types	Testing			Training Acc (%)
		Acc (%)	Se (%)	Sp (%)	
SVM	Original	88.89	89.29	88.46	94.09
	Denoised	92.59	96.43	88.46	98.96
KNN	Original	86.11	87.50	84.62	100
	Denoised	88.89	87.50	90.38	100
Bagged Tree	Original	85.19	85.71	84.62	100
	Denoised	89.81	91.07	88.46	99.90

**Table 4:** Comparison of performance metrics of test sets in different time-scales based on the optimized SVM algorithm.

Time	Acc (%)	Se (%)	Sp (%)
1 s	68.89	70.71	66.92
2 s	81.48	78.57	84.62
3 s	92.59	**96.43**	88.46
4 s	**94.44**	91.07	**98.08**
5 s	**94.44**	92.86	96.15

The highest score is indicated in bold.

**Table 5:** Comparison of performance metrics of the dataset on the 5 s time-scale based on different ML Techniques.

Models	Testing			Training	Validation
	Acc (%)	Se (%)	Sp (%)	Acc (%)	Acc (%)
KNN	92.59	89.29	96.15	**100**	**93.8**
Bagged Tree	**94.44**	89.29	**100**	**100**	89.4
Decision Tree	87.04	85.71	88.46	91.70	84.6
Naive Bayes	85.19	71.43	88.46	91.08	72.6
SVM	**94.44**	**92.86**	96.15	**100**	93.4

The highest score is indicated in bold.

**Table 6:** Comparison of the accuracy of feature sets obtained by using different average sliding windows.

Average Sliding Window M × N		N					
		5		10		20	
		Training	Testing	Training	Testing	Training	Testing
**M**	5	**100**	94.44	99.59	94.44	**100**	**98.15**
	10	**100**	94.44	95.23	90.74	99.59	94.44
	25	99.38	94.44	**100**	**98.15**	95.44	90.74

The highest score is indicated in bold.

**Table 7:** Comparison of performance metrics for specific average sliding windows.

Average Sliding Window	Testing			Training	Validation
	Acc (%)	Se (%)	Sp (%)	Acc (%)	Acc (%)
5 × 20	98.15	96.43	100	100	93.4
25 × 10	98.15	96.43	100	100	92.9

**Table 8:** Comparison of classification results of atrial fibrillation.

Author	Database	Window	Methods	Performance		
				Acc (%)	Se (%)	Sp (%)
Asgari et al., 2015 [32]	MIT-BIH AFDB	30 s	Stationary wavelet transforms, SVM	97.1	97.0	97.1
Andersen et al., 2017 [33]	MIT-BIH AFDB	300 beats 30 s	Entropy, SVM	-	96.81 94.27	96.20 98.84
Bruun et al., 2017 [25]	MIT-BIH AFDB	10 s	Discrete wavelet transforms, Bagged tree	98.22	96.51	99.19
Babaeizadeh et al., 2009 [26]	633 Holter ECG records, MIT-BIH AFDB	-	Stationary first-order Markov process, DT	-	94 (MIT-BIH AFDB)	99 (MIT-BIH AFDB)
Kumar et al., 2018 [23]	MIT-BIH AFDB	1000 samples	Wavelet transform, RF	96.84	95.8	97.8
Faust et al., 2018 [34]	MIT-BIH AFDB	100 beats	BLSTM	98.51	98.32	98.67
Dang et al., 2019 [35]	MIT-BIH AFDB	100 samples	BLSTM	99.94 (Train) 98.63 (Validation) 96.59 (Test)	99.93 (Test)	97.03 (Test)
Wei et al., 2019 [37]	MIT-BIH AFDB	1 beat	Recurrence Complex Network, CNN	94.59	94.28	94.91
Xu et al., 2018 [38]	MIT-BIH AFDB	1 s	FSWT, CNN	84.85	79.05	89.99
Proposed method	MIT-BIH AFDB	5 s	FSWT, KSVM	100 (Train) 93.4 (Validation) 98.15 (Test)	96.43 (Test)	100 (Test)

## Data Availability

The data used to support the findings of this study are available from the open-access MIT-BIH Atrial Fibrillation Database.
